# Neuroprotection after Hemorrhagic Stroke Depends on Cerebral Heme Oxygenase-1

**DOI:** 10.3390/antiox8100496

**Published:** 2019-10-19

**Authors:** Sandra Kaiser, Sibylle Frase, Lisa Selzner, Judith-Lisa Lieberum, Jakob Wollborn, Wolf-Dirk Niesen, Niels Alexander Foit, Dieter Henrik Heiland, Nils Schallner

**Affiliations:** 1 Department of Anesthesiology and Critical Care Medicine, Medical Center—University of Freiburg, 79106 Freiburg, Germany; sandra.kaiser@uniklinik-freiburg.de (S.K.); lisa.selzner@uniklinik-freiburg.de (L.S.); judith-lisa.lieberum@uniklinik-freiburg.de (J.-L.L.); jakob.wollborn@uniklinik-freiburg.de (J.W.); 2 Faculty of Medicine, University of Freiburg, 79106 Freiburg, Germany; 3 Department of Neurology, Medical Center—University of Freiburg, 79106 Freiburg, Germany; sibylle.frase@uniklinik-freiburg.de (S.F.); wolf-dirk.niesen@uniklinik-freiburg.de (W.-D.N.); 4 Department of Neurosurgery, Medical Center—University of Freiburg, 79106 Freiburg, Germany; niels.foit@uniklinik-freiburg.de (N.A.F.); dieter.henrik.heiland@uniklinik-freiburg.de (D.H.H.)

**Keywords:** cerebrovascular stroke, heme oxygenase, microglia, neuroprotection, subarachnoid hemorrhage

## Abstract

(1) Background: A detailed understanding of the pathophysiology of hemorrhagic stroke is still missing. We hypothesized that expression of heme oxygenase-1 (HO-1) in microglia functions as a protective signaling pathway. (2) Methods: Hippocampal HT22 neuronal cells were exposed to heme-containing blood components and cell death was determined. We evaluated HO-1-induction and cytokine release by wildtype compared to tissue-specific HO-1-deficient (*LyzM-Cre.Hmox1 ^fl/fl^*) primary microglia (PMG). In a study involving 46 patients with subarachnoid hemorrhage (SAH), relative HO-1 mRNA level in the cerebrospinal fluid were correlated with hematoma size and functional outcome. (3) Results: Neuronal cell death was induced by exposure to whole blood and hemoglobin. HO-1 was induced in microglia following blood exposure. Neuronal cells were protected from cell death by microglia cell medium conditioned with blood. This was associated with a HO-1-dependent increase in monocyte chemotactic protein-1 (MCP-1) production. HO-1 mRNA level in the cerebrospinal fluid of SAH-patients correlated positively with hematoma size. High HO-1 mRNA level in relation to hematoma size were associated with improved functional outcome at hospital discharge. (4) Conclusions: Microglial HO-1 induction with endogenous CO production functions as a crucial signaling pathway in blood-induced inflammation, determining microglial MCP-1 production and the extent of neuronal cell death. These results give further insight into the pathophysiology of neuronal damage after SAH and the function of HO-1 in humans.

## 1. Introduction

Neuronal injury following subarachnoid hemorrhage, a subtype of hemorrhagic stroke that bears a high mortality rate, can be profound [1,2]. The pathology of the underlying death of neuronal cells is complex and remains poorly understood. Cerebral vasospasm causing secondary ischemia has been identified as a main contributor to neuronal cell death [3]. However, clinical data show that resolution of vasospasm does not necessarily correlate with improved clinical outcome [4]. The role of the inflammatory response to blood deposition within the brain continues to be controversial as both detrimental and beneficial roles of the neuroinflammatory response after hemorrhage have been described [5,6]. Unarguably, extravascular blood components, especially heme-containing erythrocytes, pose a direct toxic challenge to the delicate milieu of the brain [7,8]. This makes the amount of deposited blood and the inflammatory response by microglia as the innate immune cells of the brain crucial determinants regarding the extent of injury.

We have recently demonstrated that the heme oxygenase enzyme (HO) system determines the rate of microglial erythrophagocytosis and heme removal [9]. Heme oxygenase (HO) enzymes degrade heme to form biliverdin, iron, and carbon monoxide (CO) gas. The inducible HO-1 isoform is ubiquitously expressed, while the constitutively expressed HO-2 isoform can only be found in some tissues, including the brain and endothelium, to regulate vasomotor tone. HO-1 confers cytoprotection in various disease models including visceral organ injury [10], heart ischemia [11], and brain injury [12]. Regarding the brain, HO-1—unlike HO-2—is heavily upregulated in glial cells following injury [13,14,15]. The byproduct of heme degradation, CO gas, has neuroprotective effects itself when given exogenously [16] and can compensate endogenous HO deficiency [9].

These findings indicate that direct neurotoxicity after hemorrhage by erythrocyte extravasation, blood clearance, and the inflammatory response by microglia are the three key components that determine neuronal cell death and brain injury.

In this study, we aimed to explore how microglia-driven inflammation contributes to neuronal injury following hemorrhage using mechanistic in vitro studies correlated with observational data in patients with SAH, hypothesizing that HO-1 would determine the inflammatory response and ultimately neuronal outcome.

## 2. Materials and Methods

### 2.1. Culture of Cell Lines and Primary Microglia

Hippocampal murine neuronal HT22 cells and BV-2 microglia cells were incubated in DMEM containing penicillin–streptomycin and 10% FBS in a humidified atmosphere with 5% CO_2_. Cells were seeded into 6-well plates at a density of 200,000 cells two days beforehand for individual experiments. *LyzM-Cre* mice were obtained from the Jackson Laboratory, Bar Harbor, ME, USA, (#004781). A cell-specific HO-1 knockout in microglia (*LyzM-Cre.Hmox1 ^fl/fl^*) was achieved by crossing *Hmox1 ^fl/fl^* mice (Riken Bio Resource Center, 3-1-1 Koyadai, Tsukuba, Ibaraki, Japan, RBRC03163) with mice expressing Cre recombinase under the lysozyme (Lyz) promoter. Animals were fed with standard rodent diet ad libitum while kept on a 12 h light/12 h dark cycle. Primary microglia (PMG) from *Hmox1 ^fl/fl^* or *LyzM-Cre.Hmox1 ^fl/fl^* were isolated from mice at P5 to P7 by enzymatic neural dissociation (Papain Neural Dissociation Kit; Miltenyi Biotec, 51429 Bergisch Gladbach, Germany) and in vitro cultivated in a mixed glia culture. In brief, pups were decapitated, and mouse brains were enzymatically dissociated according to the manufacturer’s instructions. The resulting mixed glia culture containing astrocytes and microglia was cultivated in DMEM containing penicillin–streptomycin, 10% FBS, and M-CSF (10 ng/mL) in a humidified atmosphere with 5% CO_2_. After 1 week of cultivation, cell culture plates were shaken at 200 rpm for 2 h every 2 to 3 days. Floating microglia were collected from the supernatant and seeded onto 6-well plates for experiments at a density of 2 × 10^5^ cells per well. Microglial phenotype was confirmed by CD11b staining.

### 2.2. Cell Treatment

HT22 cells were incubated at the indicated concentrations and durations with whole murine blood that was drawn from the mandibular vein of a C57BL/6 WT animal and washed with PBS. Furthermore, HT22 cells were incubated with hemoglobin (Sigma-Aldrich H7379, Taufkirchen, Germany) and hemin (Sigma-Aldrich H9039, Taufkirchen, Germany) as indicated in the individual experiments. Concentrations of hemoglobin and heme were used to correspond to the number of RBCs (1 × 10^6^, 10^7^, 10^8^) in 0.1, 1, and 10 µL mouse blood. Calculations were done as follows: 1 μL of murine blood contains 1 × 10^7^ RBC; 100 mL of murine blood contains 13 g of hemoglobin; MW of hemoglobin 64.5 g/mol (0.2 mol per 100 mL blood). As calculated for 5 mL cell culture medium used per cell culture plate well corresponding to 0.1, 1, and 10 µL blood per well led to the concentrations of 0.04, 0.4, and 4 mM hemoglobin being used in the experiments. Around 1 × 10^9^ molecules heme per RBC (2.5 × 10^8^ hemoglobin molecules per RBC, 1 hemoglobin contains 4 heme complexes), 1 RBC contains 1.6 × 10^−15^ mol heme (6 × 10^23^ molecules > 1 mol); 1 × 10^7^ RBC contain 1.6 × 10^−8^ mol > 16 nmol; corresponding end concentrations were 32 nM to 3.2 µM with an extended dose range used in the experiments. Mitochondria and mitochondrial DNA were isolated (Abcam, ab65321, Cambridge, UK) and incubated with HT22 cells as indicated. The formyl peptide receptor agonist WKYMVM was purchased from Tocris (1799). For co-culture experiments, BV-2 microglia cells were incubated with either blood or equivalent amounts of sterile latex beads (7 µm, Sigma-Aldrich, Taufkirchen, Germany) for 24 h. Medium from untreated microglia served as control medium. Microglia cell supernatant was harvested and centrifuged. HT22 cells were then incubated with 1:1 diluted conditioned microglia media for 24 h (cell growth, microscopy) or with microglia media conditioned with 10 µL blood followed by exposure to the indicated amount of blood to study neuronal apoptosis. Cells in which we intended to study the role of CO were transferred to an airtight, humidified chamber (C-Chamber; Biospherix, Parish, NY, USA) after starting erythrocyte exposure and next exposed to 250 ppm CO and 5% CO_2_ for various durations. This was controlled by an automated gas delivery system (Oxycycler; Biospherix, Parish, NY, USA). Cells were then harvested using trypsin for downstream analysis.

### 2.3. Annexin V/Propidium Iodide (PI) Staining and Flow Cytometry

Staining with annexin V–FITC and propidium iodide (PI) (Becton Dickinson, Heidelberg, Germany, PI was detected in the PE channel) and flow cytometric analyses (Attune, Applied Biosystems, Life Technologies GmbH, Darmstadt, Germany) were performed following the manufacturers’ instructions. Dead cells were gated as the sum of both annexin V highly positive/PI negative together with annexin V highly positive/PI positive.

### 2.4. Western Blot HO-1

Cells were washed with PBS and lysed in radioimmunoprecipitation assay (RIPA) buffer by shaking with 14,000 rpm for 10 min at 4 °C. Equal amounts of protein were separated on a 10% TGX stain-free polyacrylamide gel (Bio-Rad #161-0183, Feldkirchen, Germany) and transferred with a Trans-Blot Turbo transfer system (Bio-Rad) on a polyvinylidene difluoride (PVDF) membrane (Bio-Rad #1704156, Feldkirchen, Germany). Membranes were blocked with 5% skim milk in Tween-20/TBS and incubated with the recommended dilution of specific antibodies (HO-1; 1:1.000; Abcam, Cambridge, UK) overnight at 4 °C. Membranes were then incubated with the corresponding secondary antibody for chemiluminescence detection and developed on a Fusion FX imaging system.

### 2.5. Light Microscopy and Cell Quantification

HT22 cell growth after incubation with conditioned microglia media was analyzed using a Neubauer cell counting chamber. We calculated the number of cells per 6-well after 24 h of incubation with conditioned medium.

### 2.6. Bead-Based Flow Cytometry of Cell Supernatants

PMG from *Hmox1 ^fl/fl^* or *LyzM-Cre.Hmox1 ^fl/fl^* were incubated with blood +/− CO at the indicated concentration and time. Cell supernatant was harvested and analyzed using bead-based flow cytometry as per manufacturer’s instructions (552364, Mouse Inflammation Kit, Becton Dickinson GmbH, Heidelberg, Germany). The following targets were measured on a BD Fortessa flow cytometer: IL-6, IL-10, MCP-1, IFN-γ, TNF, IL-12p70.

### 2.7. Human Sample Collection and Analysis

Cerebrospinal fluid (CSF) and blood samples were acquired from SAH patients admitted to the ICU on day 1 and day 7. Inclusion criteria were (1) patients over the age of 18 years with aneurismal SAH defined by initial CT imaging or by the presence of blood and xanthochromia in the CSF, (2) symptom onset less than 24 h in duration, and (3) external ventricular drain placement as a therapeutic or diagnostic intervention. Exclusion criteria were (1) death within 24 h after admission, (2) admission later than 24 h after symptom onset, (3) evidence of subdural or epidural hemorrhage on imaging, or (4) evidence of meningitis/ encephalitis on imaging.

RNA from CSF cells was isolated with TRIzol, concentrated by spin-column purification (RNeasy Micro Kit, Qiagen, Hilden, Germany) and cDNA was acquired by reverse transcription (iScript cDNA Synthesis Kit, Bio-Rad, Feldkirchen, Germany). The mRNA level of HO-1, HO-2, and BLVRA were analyzed by real-time PCR (PowerUP SYBR Green Master Mix, Applied Biosystems, Life Technologies GmbH, Darmstadt, Germany) with RPL13A as a reference gene. As a reference population to calculate relative changes in mRNA level, we obtained intraoperative CSF samples from patients with shunt implantation and without intracerebral hemorrhage.

Primer sequences were:

1. HMOX-1 (Accession Number: NM_002133)
  Forward: GTGATAGAAGAGGCCAAGACTG
  Reverse: GAATCTTGCACTTTGTTGCTGG
2. HMOX-2 (Accession Number: NM_002134)
  Forward: AAGAGAGGATCGTGGAGGAG
  Reverse: CTTTGTCTTGTTCAGCAGCG
3. BLVRA (Accession Number: NM_000712)
  Forward: AGCTTTCTCTTGTGTCTGCC
  Reverse: ACATTCTCCAAGGACCCAGA
4. RPL13A (Accession Number: NM_012423)
  Forward: CGCTGTGAAGGCATCAACATTTC
  Reverse: GCTGTCACTGCCTGGTACTTC

Blood samples were collected into tubes with RNA stabilizing solution (Tempus Blood RNA Tubes; Applied Biosystems). RNA from peripheral blood cells was isolated using spin column purification (Tempus Spin RNA Isolation Kit; Applied Biosystems). The mRNA levels were analyzed as described above.

Bilirubin, hemoglobin, and protein content in the CSF were analyzed by spectrometry as described previously [17]. In brief, absorbance at 340, 415, and 460 nm was measured and bilirubin (c1), hemoglobin (c2), and protein (c3) content was calculated using the following formulas:

A_340_ = 0.012 × c1 + 0.0015 × c2 + 0.000054 × c3 (1)

A_415_ = 0.049 × c1 + 0.0069n × c2 + 0.000016 × c3 (2)

A_460_ = 0.083 × c1 + 0.0006 × c2 + 0.000007 × c3 (3)

HO-1 mRNA level and bilirubin content in SAH patients were compared to radiographic subarachnoid hematoma volume (HV). HV was calculated using OsiriX software as follows: cisternal hematoma areas (specifically, the prepontine cistern, the interpeduncular cistern, and the ambient cisterns) at the level of the caudal pons extending superiorly to the midbrain were measured over a vertical distance of 15 mm in adjacent axial CT slices and hematoma volumes were calculated using the measured areas and the known slice thickness. If present, ventricular hematoma (lateral, third, and fourth ventricles) as well as parenchymal and subdural hematoma volumes were measured and added to the cisternal hematoma volumes in order to give an accurate representation of the total intracranial hematoma burden. Functional outcome was evaluated on admission and discharge using a previously described scoring system (mRS, modified Rankin scale) [18]. This was then correlated with HO-1 mRNA level in relation to the measured hematoma size.

### 2.8. Statistics

Data were analyzed with a computerized statistical program (GraphPad Prism Version 7, GraphPad Software, San Diego, CA, USA). Results are presented as means (±SD). Two groups were compared with Student’s *t*-test, while multiple groups were compared with one-way ANOVA with post hoc Bonferroni multiple comparison. In vitro experiments were repeated at least three times. A *p*-value smaller than 0.05 was considered to be statistically significant.

A total of 46 patients with SAH were analyzed. Sample size calculation (chi-squared test contingency table; HO-1 vs. mRS groups; expected effect size w = 0.55; alpha = 0.05; power = 0.9) yielded a minimum group size of 42. The modified Rankin scale (mRS) was calculated on the day of admission and discharge for each patient. Patients were divided into two groups using the linear regression equation obtained for the whole patient population (Y = 3.646X + 14.69), defining either high or low HO-1 mRNA level for each individual patient in relation to the total subarachnoid hematoma volume quantified on the initial CT scan upon admission. Spearman rank correlation was used to describe the relationship between CSF HO-1 mRNA level and hematoma size. Sampling distributions into the categories were evaluated using chi-squared test. Frequencies of observed mRS at discharge were compared to high or low HO-1 mRNA level.

### 2.9. Study Approval

All procedures involving the animals were approved by the Committee of Animal Care of the University of Freiburg (Permit No. G15/61). They were conducted and reported in accordance with the ARRIVE guidelines.

Patients were studied under a protocol for acquiring CSF and blood samples that was approved by the Institutional Ethics Committee of the University of Freiburg (Protocol No. 293/15) and provided informed consent from the patient, legal guardian, or by proxy. The trial was registered with the German Clinical Trials Register (Trial-ID DRKS00008981; Universal Trial Number U1111-1172-6077).

## 3. Results

### 3.1. Whole Blood and Hemoglobin, but Not Hemin, Induce Neuronal Cell Death in Vitro

**Figure 1:**
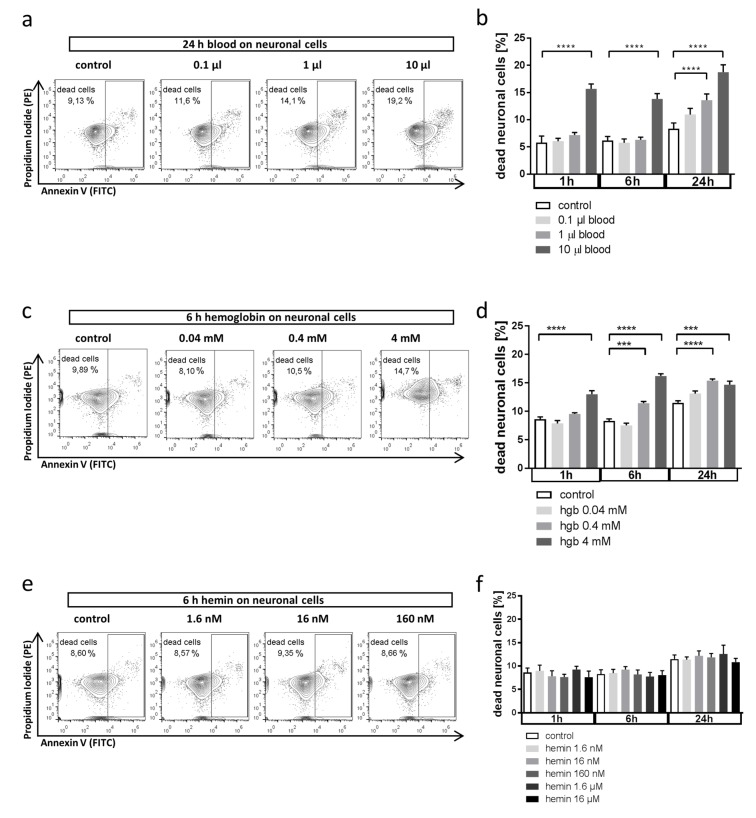
Effect of whole blood and erythrocyte components on neuronal cell death. (**a**,**b**) Effect of whole blood incubation on neuronal cell death in HT22 cells in vitro analyzed by flow cytometry after annexin V/PI staining. Cells were exposed to blood at indicated amounts (in 5 mL cell culture medium) and durations before analysis ((**a**) representative flow cytometry plots; propidium iodide (PI) was detected in the PE channel and annexin V in the FITC channel; (**b**) quantification as % dead cells from total of *n* = 6 experiments). **** *p* < 0.0001 control vs. 10 µL blood 1 h, **** *p* < 0.0001 control vs. 10 µL blood 6 h, **** *p* < 0.0001 control vs. 1 µL 24 h, **** *p* < 0.0001 control vs. 10 µL 24 h. (**c**,**d**) Effect of hemoglobin exposure on neuronal cell death in HT22 cells in vitro analyzed by flow cytometry after annexin V/PI staining. Cells were exposed to hemoglobin at indicated concentrations corresponding to the volumes of blood used in (**a**,**b**) ((**c**) representative flow cytometry plots; (**d**) quantification as % dead cells from total of *n* = 6 experiments). **** *p* < 0.0001 control vs 4 mM hemoglobin 1 h, *** *p* = 0.0002 control vs. 0.4 mM hemoglobin 6 h, **** *p* < 0.0001 control vs 4 mM hemoglobin 6 h, **** *p* < 0.0001 control vs. 0.4 mM hemoglobin 24 h, *** *p* = 0.0002 control vs 4 mM hemoglobin 24 h. (**e**,**f**) Effect of hemin on neuronal cell death in HT22 cells in vitro analyzed by flow cytometry after annexin V/PI staining. Cells were exposed to hemin at indicated concentrations corresponding to the volumes of blood used in (**a**,**b**) ((**e**) representative flow cytometry plots; (**f**) quantification as % dead cells from total of *n* = 6 experiments). *p* = n.s. for all comparisons.

We first aimed to answer the questions whether blood exerts direct toxic effects on neuronal cells and which specific blood components are responsible for these effects. We analyzed in vitro neuronal cell death by incubating HT22 cells with whole blood, free hemoglobin, or hemin. We observed a time- and dose-dependent increase in neuronal cell death in response to blood (Figure 1a,b). A similar kinetic was observed with free hemoglobin in doses equivalent to those present in whole blood (Figure 1c,d). Free heme is rapidly oxidized to hemin, which is less toxic than reduced heme. Consequently, neuronal cell death was only induced when using supraphysiological doses of hemin (Figure 1e,f). In summary, our initial results provide in vitro evidence of direct neurotoxicity following blood deposition, more specifically, by heme-containing hemoglobin, as it would happen after hemorrhagic brain injury.

### 3.2. Neuronal Apoptosis Is Not Affected by Mitochondrial DAMPs

**Figure 2:**
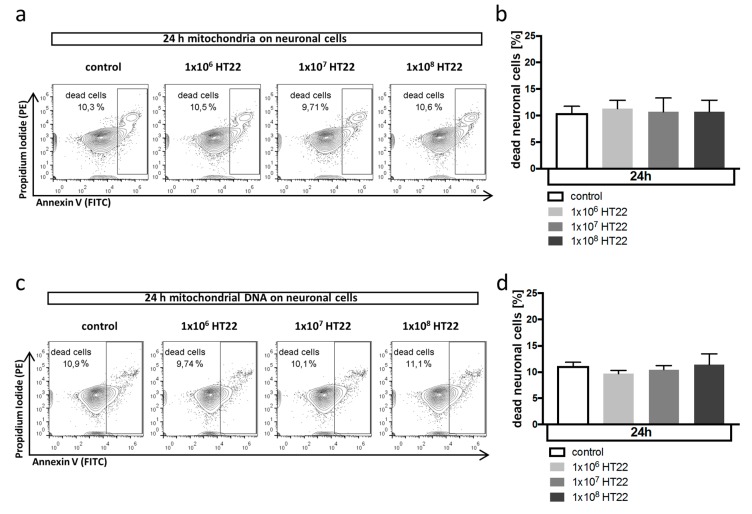
Mitochondrial DAMP pathway does not affect neuronal cell death. (**a**,**b**) Effect of mitochondria isolated from HT22 cells on neuronal cell death in HT22 cells in vitro analyzed by flow cytometry after annexin V/PI staining. Cells were exposed to mitochondria isolated from the indicated number of cells ((**a**) representative flow cytometry plots; (**b**) quantification as % dead cells from total of *n* = 6 experiments). *p* = n.s. for all comparisons. Propidium iodide (PI) was detected in the PE channel and annexin V in the FITC channel. (**c**,**d**) Effect of mitochondrial DNA isolated from HT22 cells on neuronal cell death in HT22 cells in vitro analyzed by flow cytometry after annexin V/PI staining. Cells were exposed to mitochondrial DNA isolated from the indicated number of cells ((**c**) representative flow cytometry plots; (**d**) quantification as % dead cells from total of *n* = 6 experiments). *p* = n.s. for all comparisons.

During neuronal cell death after hemorrhagic brain injury, dying neurons release intracellular components not usually present in the extracellular space, including mitochondria and mitochondrial DNA. Due to their prokaryotic origin, they can serve as damage-associated molecular patterns (DAMPs), inducing cellular injury and cell death. We therefore explored next the hypothesis that mitochondrial DAMPs could induce neuronal cell death themselves. However, incubation of HT22 cells with mitochondria isolated from neuronal cells (Figure 2a,b) or with mitochondrial DNA from the indicated number of HT22 cells (Figure 2c,d) did not directly induce neuronal cell death, suggesting that the damaging effect of DAMPs is indirect by activation of the innate immune response, namely, microglia cells within the brain that trigger a damaging inflammatory response.

### 3.3. Microglia Express HO-1 in Response to Blood Exposure While Sustaining Viability

**Figure 3:**
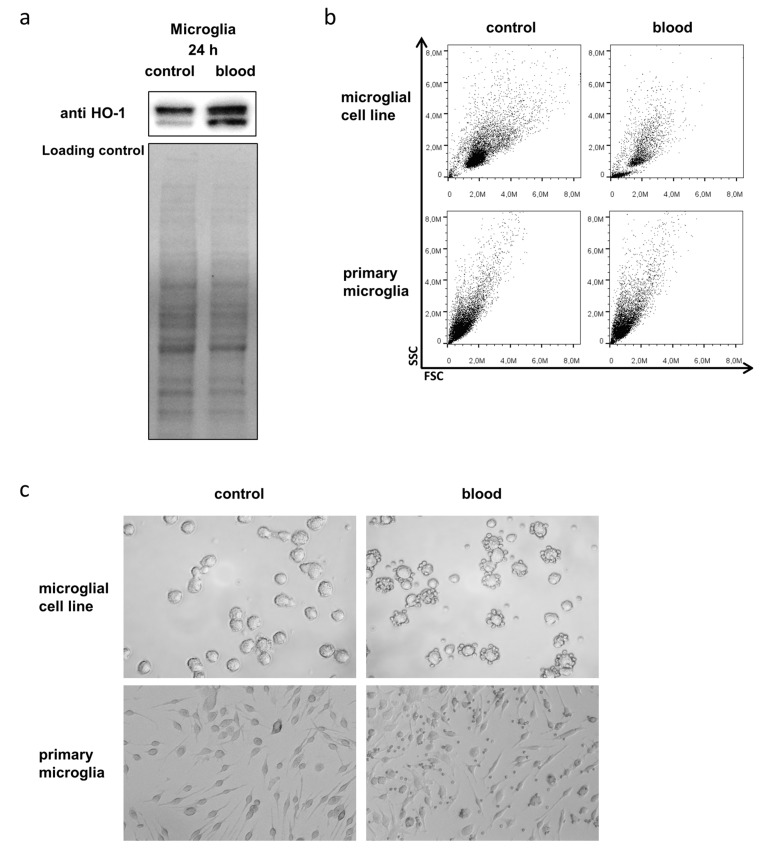
Microglial HO-1 expression and viability in response to blood exposure. (**a**) Representative Western blotting shows induction of HO-1 protein expression in BV-2 microglia in response to exposure to 10 µL blood for 24 h. Double bands are due to antibody reactivity against the truncated form of HO-1. Lower panel shows corresponding loading control using total protein staining. (**b**) Representative flow cytometry FSC/SSC dot plots of BV-2 microglia (upper panel) and primary microglia (lower panel) without and with blood exposure (10 µL accordingly). (**c**) Representative light microscopy images of BV-2 microglia and primary microglia before (left panel) and after blood exposure (right panel; 10 µL accordingly; after washing step to remove erythrocytes). Unchanged morphology, size, and granularity of microglia after exposure to blood indicated unchanged viability.

Microglia are the immunocompetent cells of the brain with myeloid origin, capable of inducing aforementioned inflammatory response in the brain. We therefore wanted to explore the role of microglia in neuronal damage after hemorrhage and asked the question whether their activation has to be considered detrimental or beneficial for neurons exposed to blood. As we have previously defined a protective role for microglial HO-1 in brain injury, we aimed to explore changes of microglial HO-1 expression in response to blood exposure. Western blot analysis showed that the cytoprotective and anti-inflammatory enzyme HO-1, which is responsible for heme degradation, was strongly induced in microglia after blood exposure (Figure 3a). Considering the detrimental effect blood exerted on neuronal cells, we also analyzed how microglial viability was affected by blood exposure. Flow cytometry (Figure 3b) and light microscopy (Figure 3c) controls demonstrated that microglia sustained normal viability and did not undergo significant blood-induced cell death. These findings indicated that microglial HO-1 is in fact involved in the injury response following hemorrhage.

### 3.4. Blood-Conditioned Medium from Microglia Exerts Pro-Survival Signaling in Neuronal Cells

**Figure 4:**
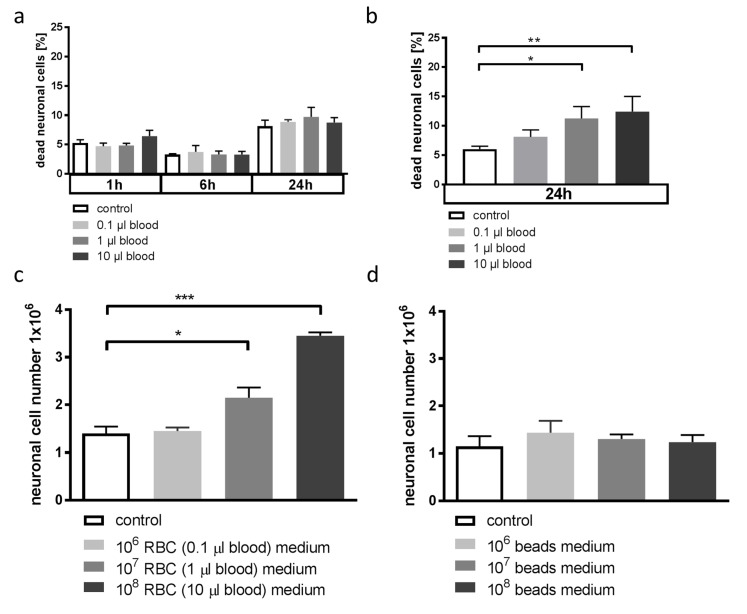
Effect of conditioned microglia medium on neuronal cell death and growth. (**a**) Quantification of flow cytometry plots after annexin V/PI staining of HT22 cells incubated with BV-2 microglia medium conditioned with 10 µL blood and then exposed to blood at the indicated amounts. Microglia medium was first conditioned as described in the methods. Conditioned medium was added to HT22 cells and cells were then exposed to blood. The % neuronal dead cells from total of *n* = 6 experiments. *p* = n.s. for all comparisons. (**b**) Quantification of flow cytometry plots after annexin V/PI staining of HT22 cells incubated with BV-2 microglia medium incubated with 10^8^ sterile latex beads and then exposed to blood at the indicated amounts. Microglia medium was first conditioned with beads as described in the methods. Conditioned medium was added to HT22 cells and cells were then exposed to blood. The % neuronal dead cells from total of *n* = 3 experiments. * *p* = 0.0133 control vs. 1 µL blood, ** *p* = 0.0031 control vs. 10 µL blood. (**c**) Microscopic cell count of neuronal HT22 cells incubated with blood-conditioned microglia medium. Microglia were exposed to the indicated amount of blood for 24 h. Conditioned medium was then added to HT22 cells for 24 h before analysis. * *p* = 0.0325 untreated medium vs. 10^7^ RBC-conditioned medium, *** *p* = 0.0007 untreated medium vs. 10^8^ RBC-conditioned medium, *n* = 6 experiments. (**d**) Microscopic cell count of neuronal HT22 cells incubated with bead-conditioned microglia medium. Microglia were exposed to the indicated amount of sterile latex beads for 24 h. Conditioned medium was then added to HT22 cells for 24 h before analysis. *p* = n.s. for all comparisons.

We next aimed to answer the question whether the observed changes in microglial HO-1 expression and concomitant changes in the inflammatory response would be beneficial or detrimental in our in vitro setting. Therefore, we studied whether the changes in microglial HO-1 expression would influence the susceptibility of neuronal cells to blood-induced injury. When cell medium from microglia that had been incubated with blood was added to neuronal cells, these cells were less susceptible to blood-induced cell death compared to cells incubated with unconditioned medium (Figure 4a). When microglia were conditioned with sterile latex beads instead of blood, the microglia supernatant did not prevent neuronal cell death by blood (Figure 4b). Moreover, incubation of neuronal cells with blood-conditioned microglia media strongly reduced neuronal cell turnover during cultivation. This reduction in neuronal cell loss was not seen when microglia were incubated with sterile beads instead of blood (Figure 4c,d). In summary, these experiments demonstrated that blood exposure of microglia indirectly activated protective and pro-survival signaling in neuronal cells via pathways yet to be determined. In the next set of experiments, we aimed to explore the changes in microglial cytokine expression related to HO-1 as a potential pathway of microglial medium-induced neuroprotection.

### 3.5. Blood Exposure Leads to a HO-1-Dependent Shift in the Microglial Cytokine Profile Influencing Neuronal Survival

**Figure 5:**
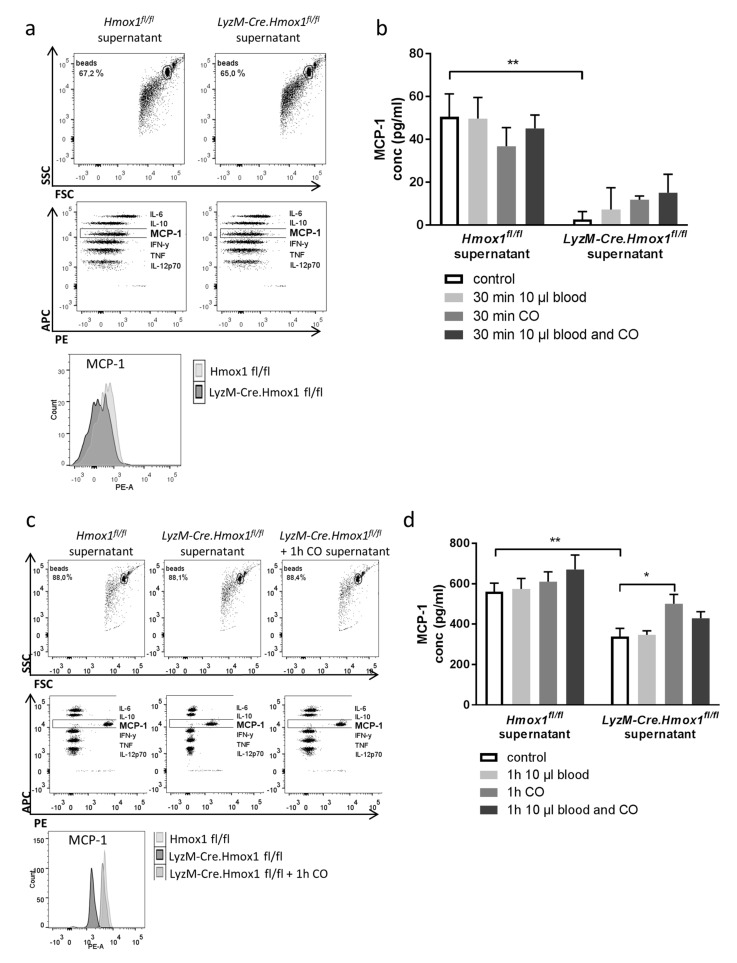

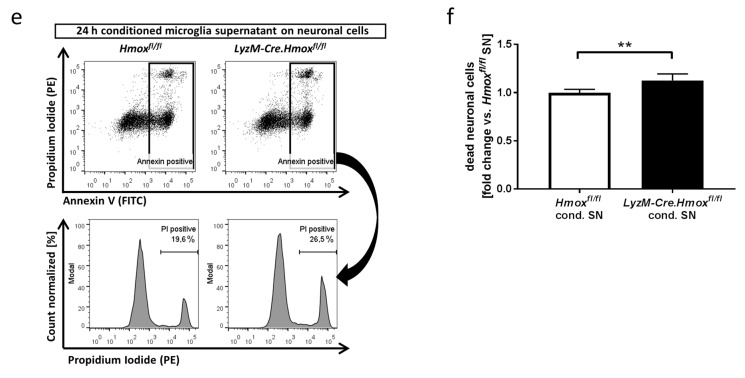
HO-1 dependent shift in microglial cytokine release after blood exposure. (**a**) Representative flow cytometry plots analyzing the cytokine secretion by *Hmox1 ^fl/fl^* or *LyzM-Cre.Hmox1 ^fl/fl^* microglia after 30 min of blood and CO exposure, Cytometric Bead Array (CBA) Mouse Inflammation Kit with IL-6, IL-10, MCP-1, IFN-γ, TNF, and IL-12p70 (**b**) Flow cytometric determination of microglial MCP-1 secretion by *Hmox1 ^fl/fl^* or *LyzM-Cre.Hmox1^fl/fl^* microglia +/− blood and CO 250 ppm, 30 min. ** *p* = 0.009 for control *Hmox1 ^fl/fl^* vs. *LyzM-Cre.Hmox1 ^fl/fl^*, *n* = 3 experiments. (**c**) Representative flow cytometry plots analyzing the cytokine secretion by *Hmox1 ^fl/fl^* or *LyzM-Cre.Hmox1 ^fl/fl^* microglia after 1 h of blood and CO exposure. (**d**) Flow cytometry of microglial MCP-1 secretion in *Hmox1 ^fl/fl^* or *LyzM-Cre.Hmox1 ^fl/fl^* microglia +/− blood and CO 250 ppm, 1 h. ** *p* = 0.001 for control *Hmox1 ^fl/fl^* vs. *LyzM-Cre.Hmox1 ^fl/fl^*, * *p* = 0.05 *LyzM-Cre.Hmox1 ^fl/fl^* control vs. CO, *n* = 3 experiments. (**e**,**f**) Effect of factors released by microglia on neuronal cell death in HT22 cells in vitro analyzed by flow cytometry after annexin V/PI staining. HT22 cells were exposed to 10 µL blood and cultivated for 24 h in 50% supernatant of cultured microglia isolated from either *Hmox ^fl/fl^* or *LyzM-Cre.Hmox ^fl/fl^* mice and conditioned with 10 µL blood exposure for 1 h. ((**e**) representative flow cytometry plots (cond = conditioned; SN = supernatant; PI = propidium iodide; propidium iodide (PI) was detected in the PE channel and annexin V in the FITC channel); (**f**) quantification as fold change vs. *Hmox ^fl/fl^* from total of *n* = 6 experiments; dead neuronal cells = annexin V and PI-positive cells; ** *p* = 0.0026).

With evidence that mediators secreted by microglia critically determine neuronal cell death, we now aimed to characterize the cytokine profile after exogenous CO exposure and relate it to HO-1 expression with subsequent endogenous production of CO. For this purpose, we incubated microglia from wildtype and HO-1-deficient animals (*LyzM-Cre.Hmox1^fl/fl^*) with blood and CO. Within a comprehensive panel of inflammatory cytokines relevant in central nervous disease, production in wildtype microglia differed from *LyzM-Cre.Hmox1^fl/fl^* microglia specifically in the amount of secreted monocyte chemoattractant protein 1 (MCP-1) (Figure 5a–d), while other inflammatory cytokines remained more or less unchanged. Additionally, we found genotype related differences at baseline and after CO and blood exposure in *LyzM-Cre.Hmox1^fl/fl^* microglia. Furthermore, deficient MCP-1 secretion in *LyzMCre-Hmox1 ^fl/fl^* microglia was partly compensated by exposure to CO. To further analyze the effect of this HO-1 dependent secretion, neuronal HT22 cells were exposed to blood and supernatant of cultured microglia which were isolated from either *Hmox ^fl/fl^* or *LyzM-Cre.Hmox ^fl/fl^* mice and conditioned with blood. FACS analysis showed increased cell death of HT22 cells cultivated with microglia supernatant of HO-1-deficient mice compared to cultivation with *Hmox ^fl/fl^* microglia supernatant (Figure 5e,f). Therefore, with these experiments we showed that HO-1 with endogenous CO production plays a crucial role in secretion of the pro-survival factor MCP-1 by microglia, thus influencing neuronal protection after blood exposure.

### 3.6. Neuronal Outcome in SAH Patients Correlates with Relative HO-1 Expression in the Cerebrospinal Fluid

**Figure 6:**
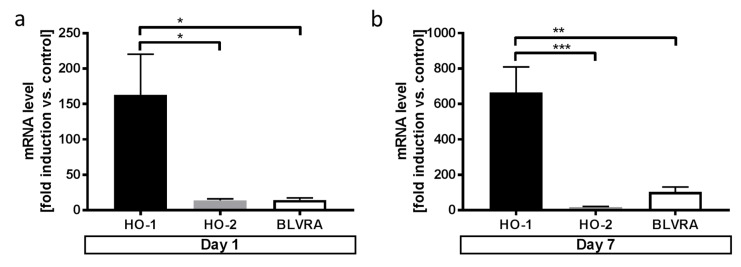
Comparison of HO-1, HO-2, and BLVRA mRNA levels in the cerebrospinal fluid (CSF) after SAH. (**a**) HO-1 vs. HO-2 and BLVRA mRNA level in cells of the CSF in SAH patients on day 1 (following SAH (Student’s *t*-test; * *p* = 0.03 HO-1 vs HO-2 and HO-1 vs BLVRA). (**b**) HO-1 vs HO-2 and BLVRA mRNA level in cells of the CSF in SAH patients on day 7 following SAH (Student’s *t*-test; *** *p* = 0.0003 HO-1 vs. HO-2; ** *p* = 0.002 HO-1 vs BLVRA).

After demonstrating the significance of microglial HO-1 regarding neuronal injury after exposure to blood in vitro, we next aimed to explore the potential in vivo role of HO-1 in patients with SAH, a subtype of hemorrhagic stroke with blood deposition onto the brain. Analyzing mRNA level in cells within the cerebrospinal fluid (CSF) of patients with SAH compared to patients without hemorrhage, we found significantly greater induction of HO-1 compared to the HO-2 isoform and the enzyme biliverdin reductase A as the downstream enzyme responsible for heme degradation (BLVRA, Figure 6a,b).

**Figure 7:**
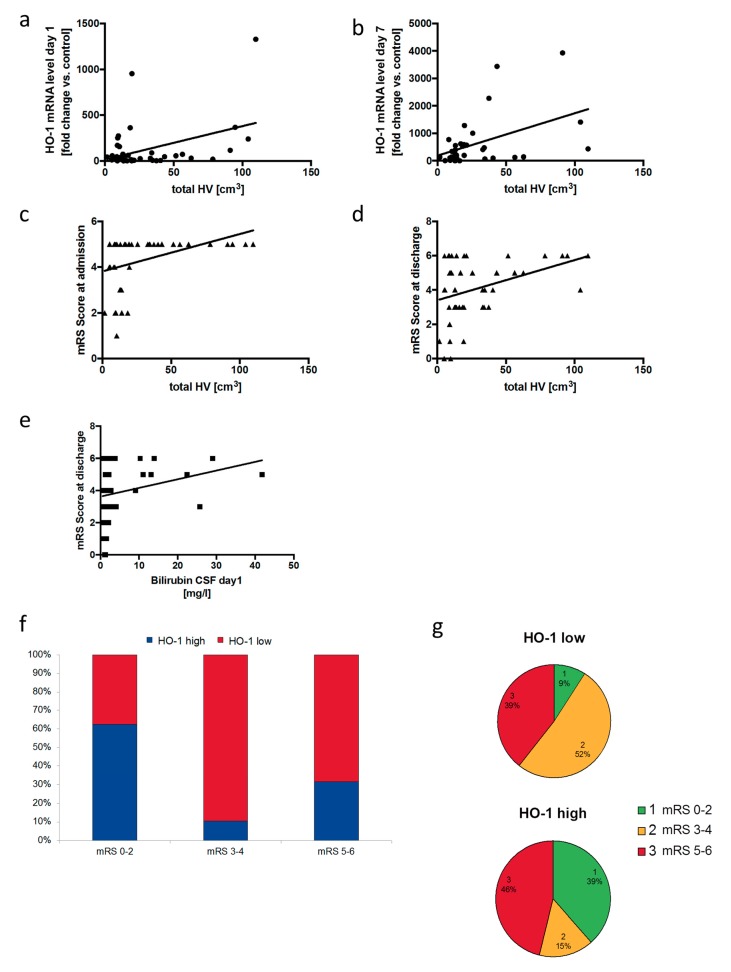
Correlation of HO-1 in the CSF of SAH patients with neurological outcome. (**a**,**b**) Correlation of HO-1 mRNA level in cells of the CSF from patients with SAH on day 1 after hemorrhage (**a**) and day 7 (**b**) with the total hematoma volume observed on the initial CT scan (Spearman correlation, *r* = 0.17, *p* = 0.3 for day 1; *r* = 0.42, *p* = 0.02 for day 7). (**c**,**d**) Correlation of the modified Rankin Scale (mRS) score from patients with SAH on admission (**c**) and discharge (**d**) with the total hematoma volume observed on the initial CT scan (Spearman correlation, *r* = 0.47, *p* = 0.001 for admission mRS; *r* = 0.32, *p* = 0.03 for discharge mRS). (**e**) Correlation of the mRS score at discharge from patients with SAH with bilirubin content in the CSF on day 1 after hemorrhage (Spearman correlation, *r* = 0.28, *p* = 0.04). (**f**,**g**) Frequencies of relative HO-1 mRNA level categories (HO-1 low vs. HO-1 high) within the observed mRS score groups in SAH patients at discharge (**f**) and distribution of the observed mRS score categories within the relative HO-1 mRNA level groups (**g**) in SAH patients at discharge (x^2^ test for sampling distribution (df = degrees of freedom); both x^2^(2) = 7.677, *p* = 0.02).

The analyses further showed that HO-1 mRNA levels in the CSF correlated with hematoma size directly not on day 1 (Figure 7a), but on day 7 (Figure 7b) after SAH. Conversely, hematoma size showed correlation with higher mRS scores, meaning more severe neurological deficits at admission and discharge (Figure 7c,d). Bilirubin content in the CSF, indicating higher cerebral heme load went along with higher mRS scores (Figure 7e). Most importantly, when dividing the patients into groups of relatively high vs. low HO-1 mRNA level in relation to the actual hematoma size using linear regression, high HO-1 mRNA level on day 1 after admission were associated with more favorable neurological outcome at discharge (Figure 7f,g). In fact, relative distribution of the favorable mRS category 0–2 (range from 0 to 2) within the HO-1 mRNA level groups was as follows: HO-1 high and mRS 0–2: 38.5% vs. HO-1 low and mRS 0–2: 9.1%. The relative distribution of HO-1 groups within the favorable mRS category 0–2 was HO-1 high 62.5% vs. HO-1 low 37.5%. To summarize the last set of experiments, these correlative human data demonstrated that high HO-1 mRNA levels in the CSF cells were associated with favorable functional outcome after SAH, supporting our in vitro findings and also suggesting a protective role for microglial HO-1 during blood-induced in vivo neuronal injury.

## 4. Discussion

Results obtained in this study demonstrate that direct neurotoxicity is exerted by blood and heme-containing hemoglobin. We provide evidence that microglial expression of HO-1 alters their inflammatory response due to change in MCP-1 production. Furthermore, we show that the HO-1-dependent cytokine release by microglia into the cellular milieu ultimately influences neuronal survival. The significance of microglial HO-1 for neuroprotection after SAH is further emphasized by our clinical data, providing evidence that adequate induction of HO-1 in the CSF influences neurological outcome in humans.

Only few studies have directly compared the extent of neurotoxicity exerted by erythrocytes or its components released after lysis within the same model. In vivo, hemolysis occurs rapidly after brain hemorrhage, within 24 h [19], releasing potentially toxic hemoglobin. Our findings are in line with previous studies suggesting that neurotoxicity is mainly due to hemoglobin [8,20]. The neurotoxic effect seen by incubation with whole blood can therefore most likely be explained by the release of hemoglobin in the course of rapid erythrocyte lysis. However, we cannot completely exclude a direct neurotoxic effect by erythrocytes themselves, as has been suggested before [21]. Free heme is a potent pro-oxidant that can cause cytotoxicity and organ damage in vivo [22]. Heme detoxification mainly occurs via the heme oxygenase system that degrades heme to biliverdin, free iron, and carbon monoxide gas. Yet, heme toxicity is challenging to study in vitro due to its rapid oxidation to hemin. Other studies have previously shown that hemin is, in fact, not as toxic as its reduced counterpart heme [23]. This was also evident in our experiments, as only doses higher than equivalent doses of whole blood and hemoglobin were able to induce neuronal cell death.

Endogenous damage-associated molecular patterns (DAMPs), such as circulating mitochondria, can cause a sterile inflammatory response after injury and are able to induce organ injury [24]. Previous studies suggest a role in acute [25] and chronic [26] brain injury. In our in vitro studies, mitochondrial DAMPs were not able to recapitulate the neurotoxic effects by blood components. Our data indicate that neuronal injury seen after hemorrhage is due to direct toxic effects of blood components and not caused by an indirect neuroinflammatory response.

HO-1 induction after hemorrhage is a powerful stress response mechanism [10] and can be beneficial following neuronal injury [12,27]. However, the mechanisms of neuroprotection via HO-1 remain poorly understood, and studies have mainly focused on its vasoactive properties [28]. HO-1 is mainly upregulated in microglia following ischemic or hemorrhagic injury, suggesting that modulation of the neuroinflammatory response via HO-1 is crucial for its neuroprotective properties [14,15,29]. Here, we provide evidence that HO-1 determines the inflammatory response in microglia. In a cytokine panel relevant in central nervous disease, microglia deficient in HO-1 expressed significantly less MCP-1. Classical pro-inflammatory cytokines such as IL-6, IFN-γ, or TNF-α remained unchanged. MCP-1, which was specifically upregulated in HO-1 expressing microglia, causes migration and proliferation of microglia without directly activating their inflammatory response [30,31], pointing towards a HO-1-driven microglial polarization towards a proliferative phenotype. Furthermore, our co-culture experiments and addition of conditioned microglia supernatant demonstrate that the cytokine release by microglia within the milieu of the central nervous system can exert pro-survival signaling, depending on their inflammatory polarization involving functional HO-1.

This might also explain why the role of the neuroinflammatory response executed by microglia in neuronal injury remains controversial: increased inflammation can, on the one hand, be harmful when inducing neuronal apoptosis [32] but, on the other hand, also be neuroprotective [5,6]. In parallel to peripheral macrophages, the fate of neurons in response to neuroinflammation most likely depends on the type of injury and also on the exact mode of microglial activation and polarization [33,34]. In the future, further analysis of inflammatory markers such as IL-1β or iNOS will help to further characterize the inflammatory response exerted by microglia during hemorrhage.

The HO-2 isoform is exclusively expressed in neurons, the testis and endothelial cells. Others have demonstrated that there is no upregulation of HO-2 following hemorrhage compared to HO-1 [13,14,15]. We found significantly less HO-2 induction compared to HO-1 in cells of the CSF. This divergence might be due to methodical (qPCR vs. immunohistochemistry) differences and to the fact that few have studied the characteristics of cells within the CSF as opposed to cells within the brain itself. Nevertheless, the fact that giving exogenous CO to HO-1-deficient microglia could not fully compensate for the changes seen in the inflammatory cytokine panel supports the idea that endogenous CO produces specific intracompartmental concentrations and time kinetics that cannot be compensated for by other sources of CO gas, i.e., HO-2 in adjacent cells. In summary, our data provide evidence that the functions characterized in this study are specific for microglial HO-1.

SAH is a subtype of hemorrhagic stroke with a young age of onset and high mortality rate [35]. As treatment options remain mostly symptomatic and clinical monitoring is often limited due to the severity of the disease, there is an urgent need for more specific treatment options and useful clinical markers predicting neurological outcome and identifying patients at risk for secondary brain injury. Many patients suffer from long-term neurological deficits due to delayed cerebral ischemia (DCI) [36]. DCI happens secondarily to cerebral vasospasm, which is usually seen over the time course of the first fourteen days after SAH. However, this doctrine has been questioned, as effective treatment of cerebral vasospasm does not improve DCI-related morbidity and functional outcome [4,37]. This suggests other factors contributing to the devastating neurological outcome seen after SAH, emphasizing the role of direct blood component toxicity and pointing towards a distinct role for the neuroinflammatory response. With our correlative patient data, we provide evidence that HO-1 mRNA level in cells of the CSF is a function of the hemorrhage size, which conversely influences neurological outcome. Measuring functional neurologic outcome by the modified Rankin scale (mRS) is a well-established clinical tool [38]. As the relative quantity of HO-1 mRNA level in relation to the actual hematoma size was associated with better short-term functional outcome, we provide compelling evidence that HO-1 mitigates the severity of injury in human SAH, with potential protective properties regarding secondary brain injury. Whether the relative quantity of HO-1 mRNA level also correlates with a lower incidence of DCI and better long-term neurological outcome remains to be elucidated.

We acknowledge several limitations for our study. First, we used in vitro cell models that do not exactly reflect the pathology in human disease. Therefore, these models strictly served to study our hypothesis on the mechanism of neuronal cell damage. Further, we were not able to look into in vitro gender-specific differences as our primary microglia cell came from mixed glia cultures from both genders. Lastly, due to limitations in the amount of biological material, we were not able to further differentiate the cells contained in the CSF responsible for HO-1 expression and blood clearance.

In conclusion, this study helps to further understand the pathophysiology in hemorrhagic injury following SAH. It further defines the significance of microglial HO-1 in the neuroinflammatory response to hemorrhage and provides evidence that the HO-1 enzyme system is of pivotal importance. Analyzing HO-1 in the CSF might serve as an early prognostic marker after SAH which, in combination with recently described serum and CSF markers [39], could potentially provide clinicians with a distinguished approach to identifying patients at risk for secondary neurologic deterioration. In the future, inducing HO-1 or mimicking HO-1 induction by applying low doses of CO gas might offer new therapeutic strategies in this devastating disease.
